# The costs of stunting in South Asia and the benefits of public investments in nutrition

**DOI:** 10.1111/mcn.12281

**Published:** 2016-05-17

**Authors:** Meera Shekar, Julia Dayton Eberwein, Jakub Kakietek

**Affiliations:** ^1^ Health, Nutrition and Population Global Practice World Bank Washington District of Columbia USA

**Keywords:** stunting, South Asia, cost‐effectiveness, nutrition interventions, economic productivity

## Abstract

South Asia is home to the largest number of stunted children worldwide: 65 million or 37% of all South Asian children under 5 were stunted in 2014. The costs to society as a result of stunting during childhood are high and include increased mortality, increased morbidity (in childhood and later as adults), decreased cognitive ability, poor educational outcomes, lost earnings and losses to national economic productivity. Conversely, investing in nutrition provides many benefits for poverty reduction and economic growth. This article draws from analyses conducted in four sub‐Saharan countries to demonstrate that investments in nutrition can also be very cost‐effective in South Asian countries. Specifically, the analyses demonstrate that scaling up a set of 10 critical nutrition‐specific interventions is highly cost‐effective when considered as a package. Most of the interventions are also very cost‐effective when considered individually. By modelling cost‐effectiveness of different scale‐up scenarios, the analysis offers insights into ways in which the impact of investing in nutrition interventions can be maximized under budget constraints. Rigorous estimations of the costs and benefits of nutrition investments, similar to those reported here for sub‐Saharan countries, are an important next step for all South Asian countries in order to drive political commitment and action and to enhance allocative efficiency of nutrition resources.

## Introduction

**Figure 1:**
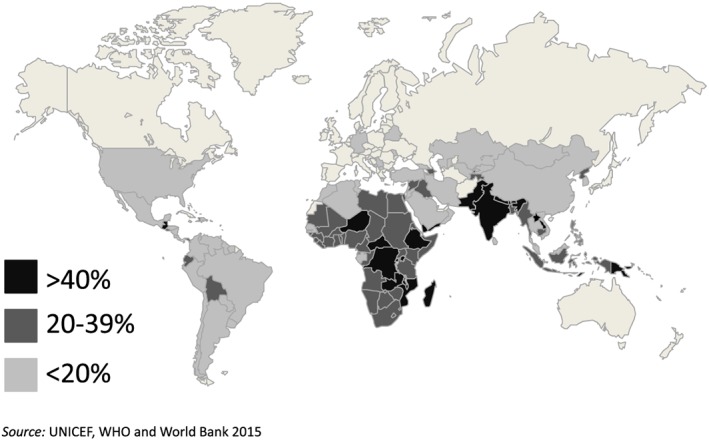
Per cent of children under 5 stunted, 2014.

South Asia is home to the largest number of stunted children worldwide (Fig. 1). Sixty‐five million or 37% of all children under 5 were stunted in South Asia in 2014 (UNICEF *et al*. 2015). Undernutrition is an underlying cause of 3.1 million child deaths annually, which accounts for 45% of all deaths in children under 5 (Black *et al*. 2013). Chronic malnutrition (stunting) alone is responsible for between 15% and 17% of all deaths in children under 5 worldwide and approximately 400 000 deaths in South Asia (Black *et al*. 2013). Undernourished children are more likely to die from common childhood illnesses such as diarrhoea, measles, pneumonia, malaria or HIV/AIDS (Black *et al*. 2013). Stunting has detrimental effects on the brain by causing deviations in the temporal sequence of brain maturation, which in turn disturb the formation of neural circuits (Udani 1992) and result in cognitive deficits (Kar *et al*. 2008). Stunted children are more likely to start school late, to repeat a grade or to drop out of school (Mendez & Adair 1999; Daniels & Adair 2004). Martorell *et al*. (2010) have shown that adults who were stunted at age 2 completed 1 less year of schooling. Adair *et al*. (2013) estimated that improving linear growth for children under 2 by one standard deviation adds about half a grade of school attainment.

Although progress has been made in reducing stunting in South Asia to 37% of children under 5, this rate is still unacceptably high. Every country in the region has observed reductions in the prevalence of stunting over the past two decades – the average annual rate of reduction is 1.7% for the region as a whole, but it varies across countries from about 1.1% in Pakistan to about 3% in the Maldives (UNICEF *et al*. 2015). However, given the current high levels of undernutrition and its slow rate of reduction during the most recent decade, most South Asian countries are unlikely to achieve the Millennium Development Goal for nutrition (target 2 of Millennium Development Goal 1) of halving, between 1990 and 2015, the proportion of children who are underweight.

Although South Asia is in many ways poised for economic transformation, the hidden burden of undernutrition slows prosperity in a number of ways. Childhood stunting costs developing countries billions of dollars in future revenue losses through reduced economic productivity, particularly through lower wages, lower physical and mental capabilities and more days away from work as a result of illness. Adult height is known to be related to productivity, and final height is determined in large part by nutrition from conception to age 2 (Behrman & Rosenzweig 2001). Not only are taller adults more physically capable and strong (which is especially important for manual labour), but greater height may also be a proxy measure of other dimensions of human capital such as social skills and cognitive ability (Hoddinott *et al*. 2013). The median increase in hourly wages per 1 cm of additional height was 4.5% from a survey of eight low‐income and middle‐income countries (Horton & Steckel 2013) and 0.55% from a study of eight high‐income countries (Gao & Smyth 2010). Horton & Steckel (2013) estimated losses from all forms of malnutrition of up to 11% of gross domestic product (GDP) in Africa and Asia each year. Adults, who were stunted as children earned 20% less than comparable adults who were not stunted (Grantham‐McGregor *et al*. 2007), were 30% more likely to live in poverty and were less likely to work in skilled labour (Hoddinott *et al*. 2011).

There is a strong and growing body of evidence identifying interventions that are effective and feasible to implement at scale (World Bank 2006; *Lancet* 2008 and 2013). The first 2 years of life – the first 1000 days between conception and age 2 – are the critical period for growth and development, while growth at older ages does not have the same effect on adult height and brain development (Black *et al*. 2013). Therefore, investments during this period of development have the highest returns (Heckman & Masterov 2004), and investing in early childhood nutrition interventions offers a window of opportunity to permanently lock‐in human capital. Indeed, the Copenhagen Consensus Center has identified nutrition interventions as one of the most cost‐effective development actions. The centre has estimated that reducing by 40% the number of children who are stunted by 2030 would return $45 for every dollar spent (Copenhagen Consensus Center 2015; Hoddinott *et al*. 2013). However, investment in nutrition in South Asia remains inadequate. The key questions that need to be answered to mobilize the political will and accelerate action to reduce stunting in South Asia are ‘How much will it cost to scale up nutrition interventions?’ and ‘What will these investments buy?’ However, at present, there are no rigorous estimates of the costs and benefits of nutrition investments in South Asia.

This article presents preliminary estimates of costs and cost‐effectiveness of investing in high‐impact evidence‐based nutrition interventions in four countries in sub‐Saharan Africa to show what investing in nutrition can buy in terms of averted deaths, cases of stunting and disability‐adjusted life years (DALYs) averted. 1A DALY is equivalent to a year of healthy life lost due to a health condition. The DALY, developed in 1993 by the World Bank, combines the years of life lost from a disease and the years of life spent with disability from the disease. By modelling cost‐effectiveness of different scale‐up scenarios, we offer insights into ways in which the impact of investing in prevention of stunting can be maximized under budget constraints.

## Methods

The preliminary results presented next are part of a larger effort to assess the cost and cost‐effectiveness of investing in nutrition in sub‐Saharan Africa. 2This work is ongoing by the World Bank with the support of the Bill and Melinda Gates Foundation. Scale‐up costs were estimated for 10 interventions in four African countries: Democratic Republic of the Congo (DRC), Mali, Nigeria and Togo.

### Interventions included and rationale for selection

The 10 nutrition‐specific interventions considered are a modified package of the interventions included in the 2008 and 2013 *Lancet* series on Maternal and Child Undernutrition (Table 1). Some interventions, such as deworming and iron fortification of staple foods, which were included in the 2008 *Lancet* series but no longer listed in the 2013 *Lancet* series, are included here as they remain relevant. Others, such as calcium supplements for women or prophylactic zinc supplementation, are excluded because delivery mechanisms are not available in developing countries and/or there are no clear World Health Organization (WHO) protocols or guidelines for large‐scale programming. In other cases, the capacity for scaling up the interventions is limited. Only those nutrition‐specific interventions that have strong evidence of effectiveness have a WHO protocol, and a feasible delivery mechanism for scale‐up is included.

**Table 1:** Ten nutrition‐specific interventions delivered primarily through health sector

Intervention	Description	Target population
Community nutrition programmes for growth promotion	Behaviour change communication focusing on optimal breastfeeding and complementary feeding practices, proper handwashing, sanitation and good nutrition practices	Mothers of children 0–59 months of age
Vitamin A supplementation	Semi‐annual doses	Children 6–59 months of age
Therapeutic zinc supplementation with ORS	As part of diarrhoea management with ORS	Children 6–59 months of age
Multiple micronutrient powders	In‐home fortification of complementary food	Children 6–23 months of age not receiving fortified complementary food
Deworming	Two rounds of treatment per year	Children 12–59 months of age
Iron–folic acid supplementation	Iron–folic acid supplementation during pregnancy	Pregnant women
Iron fortification of staple foods	Fortification of wheat flour with iron	General population
Salt iodization	Iodization of centrally processed salt	General population
Public provision of complementary food for the prevention of moderate acute malnutrition	Provision of a small amount (~250 kcal day^−1^) of nutrient‐dense complementary food for the prevention of moderate malnutrition (moderate acute malnutrition and/or moderate stunting)	Twice the prevalence of underweight (WAZ less than −2) among children 6–23 months of age
Community‐based management of severe acute malnutrition	Includes the identification of severe acute malnutrition, community or clinic‐based treatment and therapeutic feeding using ready‐to‐use therapeutic food	Incidence (estimated as two times the prevalence) of severe wasting (WAZ less than −3) plus oedema among children 6–59 months of age

ORS, oral rehydration salts; WAZ, weight‐for‐age z‐score.

### Data

Unit costs for each intervention were obtained from published sources or from in‐country stakeholders including governmental agencies, development partners and non‐governmental organizations implementing activities related to nutrition during in‐person interviews and remote follow‐up. Costing methodology followed the programme experience approach where the unit cost (cost per beneficiary) was calculated based on the budget/expenditure estimates of agencies implementing the interventions in each country. This approach generates unit cost data that capture all aspects of service delivery, including the costs of commodities, transportation and storage, personnel, training, supervision, monitoring and evaluation, relevant overhead and wastage for each intervention from actual programmes that are already in operation. Although the programme experience approach tends to yield cost estimates higher than those of the ingredient‐based approach, these estimates more accurately reflect real programmatic experience, including inefficiencies in service delivery. It should, however, be noted that the calculated costs are reported in financial or budgetary terms. They do not capture the full social resource requirements, which account for the opportunity costs of the time committed by beneficiaries accessing the services.

Programme coverage data were obtained from the most recent Demographic and Health Survey (DHS), Multiple Indicator Cluster Survey or Standardized Monitoring and Assessment of Relief and Transitions survey in each country. Nutrition status, health and other demographic data were obtained from the most recent DHS or Multiple Indicator Cluster Survey. Population data were obtained from the most recent available national census and supplemented, if needed, by the DHS data (e.g. distribution of children under 5 in different age categories: 0–6, 0–24, 6–24 and 6–50 months).

### Estimating intervention costs

The cost for the scale‐up of each intervention was calculated as follows: *Y* = (*x*_1_ + *x*_2_) − *x*_3 _where *Y* is the annual public investment required to scale‐up to full coverage; *x*_1_ is the additional total cost to scale‐up to full coverage; *x*_2_ is the additional cost for capacity development, monitoring and evaluation, and technical assistance; and *x*_3_ is the cost covered by households living above poverty line for selected interventions.

We have assumed a linear relationship between the cost and coverage with no economies or diseconomies of scale at the country level. ‘Full coverage’ is defined as 100% of the target population for all interventions except for community‐based treatment of severe acute malnutrition, for which full coverage is assumed to be 80%.

### Estimating benefits from interventions

The expected benefits from scaling up nutrition interventions are calculated in terms of (1) DALYs averted, (2) number of lives saved and (3) cases of childhood stunting averted. The projected number of lives saved and the cases of childhood stunting averted are calculated using the Lives Saved Tool (LiST), which models reductions in mortality and prevalence of under‐5 stunting resulting from changes in intervention coverage. 3For details on the tool, see http://www.livessavedtool.org/. Based on the lives saved and cases of stunting averted estimated using LiST, DALYs are estimated following the established convention of counting one life saved as 33.3 discounted DALYs averted and one case of stunting as 0.23 discounted DALY averted (Bhutta *et al*. 2009). This method discounts the DALYs at 3% following the WHO–Choosing Interventions that Are Cost‐effective (CHOICE) methodology (WHO 2003). Because of limitations of the LiST tool, estimates for the number of lives saved and DALYs averted are based on only six of the 10 interventions, 4The six interventions are community nutrition programmes for growth promotion, vitamin A supplementation, therapeutic zinc supplementation with ORS, iron–folic acid supplementation, the public provision of complementary food for the prevention of moderate acute malnutrition and community‐based management of severe acute malnutrition. and cases of childhood stunting averted are based on only four of the 10 interventions. 5The four interventions are community nutrition programmes for growth promotion, vitamin A supplementation, iron–folic acid supplementation and the public provision of complementary food for the prevention of moderate acute malnutrition. As such, the estimates presented here are likely to underestimate the number of lives saved and cases of childhood stunting averted.

Estimates of benefits were combined with information on costs to produce the cost‐effectiveness measures for each intervention as well as for the overall package of interventions. The measures for cost‐effectiveness of nutrition‐specific interventions are calculated in terms of cost per DALY averted, cost per life saved and cost per case of stunting averted. The evaluation of cost‐effectiveness ratio in terms of DALYs averted is based on societal willingness to pay thresholds based on GDP per capita, using the categorization developed by WHO‐CHOICE (WHO 2014). An intervention is considered to be ‘very cost‐effective’ if the cost per DALY averted is less than GDP per capita; it is considered to be ‘cost‐effective’ if it is between one and three times GDP per capita; and it is considered ‘not cost‐effective’ if it exceeds three times GDP per capita (WHO 2014).

In each country, we estimated the cost and impact of scaling up the high‐impact intervention at the national level (full national coverage). Given resource constraints, few countries are able to effectively scale‐up all 10 nutrition‐specific interventions to full national coverage immediately. We therefore model potential partial scale‐up scenarios with lower overall costs, based on considerations of burden of stunting, potential for impact, costs and capacity for implementation in the specific country. Three approaches are considered: (1) scaling up only in the regions with the highest burden of malnutrition; (2) scaling up only a subset of interventions nationwide; and (3) scaling up a subset of interventions only in the regions with the highest burden of malnutrition.

## Results

Table 2 provides estimates of the total costs and impacts of scaling up all 10 interventions to full coverage for the four countries. 6More detailed results are presented in Shekar *et al*. 2014, 2015a, 2015b, 2015c. The costs to scale‐up vary substantially across countries, ranging from US$13m in Togo to US$837m in Nigeria. The differences in the scale‐up costs are a result of variation in the number of beneficiaries, baseline coverage of the interventions and in unit costs. Based on the modelling results, the investments would save 115 000 DALYs in Togo, 509 000 DALYs in Mali, 2.6 million DALYs in the DRC and 6.3 million DALYs in Nigeria. For each country, the scale‐up would also save between 3000 and 180 000 lives and prevent between 60 000 and 3 million cases of stunting annually. Based on the WHO‐CHOICE criteria of cost‐effectiveness, investing in the scale‐up of the full intervention package is estimated to be very cost‐effective in each of the four countries.

**Table 2:** Costs and benefits of investing in a package of 10 (*Lancet* ±) nutrition‐specific interventions in DRC, Mali, Nigeria and Togo (US dollars)

Country region (year)	Annual public investment required	Annual estimated benefits			Cost‐effectiveness estimates			
		DALYs averted	Lives saved	Cases of stunting averted	Cost per DALY averted	Cost per life saved	Cost per case of stunting averted	WTP threshold (GDP per capita)
DRC (2015)	371 M	2.6 M	77 000	1 M	143*	4929	226	454
Mali (2015)	64 M	509 000	14 000	260 000	178*	6276	344	715
Nigeria (2014)	837 M	6.3 M	180 000	3 M	141 *	4865	292	3010
Togo (2015)	13 M	115 295	3000	60 000	127*	4635	238	636

Sources: Shekar *et al*. 2014, 2015a,2015b, 2015c.

DALY, disability‐adjusted life year; DRC, Democratic Republic of the Congo; GDP, gross domestic product; WTP, willingness to pay.

M denotes million, and B denotes billion. DALYs are discounted at 3%.

* Very cost‐effective;

† Cost‐effective; and

‡ Not cost‐effective according to WHO‐CHOICE criteria. See WHO 2014.

Most of the interventions – with the exception of the public provision of complementary food for the prevention of moderate acute malnutrition – are ‘very cost‐effective’ when considered individually (Table 3). Across all four countries, community nutrition programmes for growth promotion, vitamin A supplementation for children and therapeutic zinc supplementation with oral rehydration salts (ORS) for children are estimated to be highly cost‐effective with a cost per DALY averted of under $100 in every country. On the other hand, the public provision of complementary food is generally not as cost‐effective, with a cost per DALY averted that is much higher than the other interventions. It is above the ‘very cost effective’ threshold of GDP per capita in two of the four countries.

**Table 3:** Cost per DALY averted for nutrition‐specific interventions in four African countries (US dollars)

Intervention§	Cost/DALY averted			
	DRC (2015)	Mali (2015)	Nigeria (2014)	Togo (2015)
Community nutrition programmes for growth promotion	77*	49*	32*	40*
Vitamin A supplementation	43*	14*	50*	321*
Therapeutic zinc supplementation with ORS	71*	41*	84*	59*
Iron–folic acid supplementation§§	101*	95*	198*	236*
Public provision of complementary food for moderate acute malnutrition	478‡	803‡	738†	580‡
Community‐based treatment of severe acute malnutrition	174*	87*	172*	47*

Sources: Shekar *et al*. 2014, 2015a, 2015b, 2015c.

DALY, disability‐adjusted life year; DRC, Democratic Republic of the Congo.

DALYs are discounted at 3%.

§ Because of methodological limitations, we were not able to calculate DALYs averted, lives saved or cases of stunting averted from four interventions: multiple micronutrient powders, deworming, iron fortification of staples foods and salt iodization.

§§ DALY estimates for iron–folic acid supplementation are calculated for DALYs averted among pregnant women. They do not include the DALYs averted among children born to mothers who received these supplements.

* Very cost‐effective;

† Cost‐effective; and

‡ Not cost‐effective according to WHO‐CHOICE criteria. See WHO 2014.

Finally, we compared the full national coverage scenario with more limited scale‐up scenarios that (1) target only the regions with highest burden of stunting, 7The criteria for highest burden of stunting are country specific as follows: stunting rates greater than 45% and severe stunting greater than 25% (DRC); stunting rates greater than 40% (Mali); and 13 States with stunting rates greater than 35% (Nigeria). This scenario was not estimated for Togo. (2) scale‐up only the most cost‐effective interventions or (3) scale‐up the most cost‐effective interventions in the highest burden regions. Table 4 shows that in all four countries, scaling up all interventions except the public provision of complementary feeding was the most cost‐effective strategy (cost per DALY ranging from $79 in Togo to $109 in Nigeria). The differences between the costs per DALY averted of the scale‐up of this subset of intervention nationwide and only in high burden regions were very small. The scale‐up in high burden regions had lower cost per DALY only in Togo ($78 compared with $79 for the full national coverage scale‐up).

**Table 4:** Scale‐up scenarios for 10 nutrition‐specific interventions in four African countries (US dollars)

	
	DRC (2015)		Mali (2015)		Nigeria (2014)		Togo (2015)	
Scale‐up scenario	Total in millions	Cost per DALY averted	Total in millions	Cost per DALY averted	Total in millions	Cost per DALY averted	Total in millions	Cost per DALY averted
Full scale‐up of all 10 interventions nationwide	371	143	64	178	837	141	13	127
Full scale‐up in highest burden regions	135	173	44	212	507	257	n/a	n/a
High‐impact interventions* nationwide (excludes public provision of complementary food)	279	133	24	71	511	109	7	79
High‐impact interventions* in highest burden regions (excludes public provision of complementary food)	97	134	18	75	271	129	4	78

Sources: Shekar *et al*. 2014, 2015a, 2015b, 2015c.

DALY, disability‐adjusted life year; DRC, Democratic Republic of the Congo; n/a, not available.

All scenarios in the table are very cost‐effective according to WHO‐CHOICE thresholds (WHO 2014).

DALYs are discounted at 3%.

* Interventions include community nutrition interventions for growth promotion, vitamin A supplementation, therapeutic zinc supplementation with ORS, multiple micronutrient supplementation, deworming, iron–folic acid supplementation, iron fortification of staple foods, salt iodization, public provision of complementary food for the prevention of moderate acute malnutrition and community‐based treatment of severe acute malnutrition.

## Discussion

This paper contributes to the existing literature on the cost of scaling up nutrition interventions (World Bank 2010; Hoddinott *et al*. 2013; Bhutta *et al*. 2013). It expands it by considering the cost‐effectiveness of those investments at the country level for four African countries. Our analysis suggests that investing in the same set of interventions in South Asia is likely to be very cost‐effective.

The results show that investing in expanding the coverage of high‐impact interventions is very cost‐effective, with costs per DALY averted well below the established willingness to pay threshold. Those findings are consistent with the existing literature. Hoddinott *et al*. (2013) estimated that expanding the coverage of nutrition intervention would yield a benefit–cost ratio of 18:1. In other words, that $1 invested in nutrition produces $18' worth of benefits.

When examined individually, most of the interventions considered here are shown to be very cost‐effective according to well‐established criteria. The exception is the public provision of complementary food for the prevention of moderate acute malnutrition. Even though it is cost‐effective in two countries (DRC and Mali) and very cost‐effective in the other two (Nigeria and Togo), it is nevertheless between two and eight times more costly per DALY averted than the next least cost‐effective intervention. Therefore, in countries like the four examined here, where fiscal and capacity constraints will limit scale‐up, certain expensive interventions – such as the public provision of complementary foods – may be a lower priority. Furthermore, issues of governance, accountability and supply logistics will all put pressure on the cost and complicate the scale‐up of the public provision of complementary foods.

The cost per DALY and other outcomes were consistent in magnitude but varied slightly across the four countries. Overall, the cost per outcome was the highest in Mali ($178 per DALY averted, $6276 per life saved and $344 per case of stunting averted) and lowest in Togo ($127 per DALY averted, $4635 per life saved and $238 per case of stunting averted) and DRC ($143 per DALY averted, $4929 per life saved and $226 per case of stunting averted). The differences between countries are driven mostly by the differences in unit costs of the specific interventions and, to a much smaller extent, by the differences in the baseline coverage in and, consequently, cost of filling the coverage gaps and its impact on morbidity and mortality.

Scaling up high‐impact interventions (that is, excluding the public provision of complementary feeding) was the most cost‐effective strategy. Whether these interventions were scaled up nationwide or only in the highest burden regions did not affect their cost‐effectiveness by much. However, there was a large total cost differential between the two scenarios, and therefore in most cases, the selection between the two scale‐up scenarios hinges mainly on the availability of public resources for scaling up nutrition interventions. These results are not counterintuitive. However, they demonstrate that data‐based planning that incorporates both local need, current coverage of interventions and economic analysis is needed to ensure that investment in nutrient has the greatest possible impact. This is particularly important given the severe resource constraints faced by countries with high burdens of malnutrition.

At present, rigorous country estimates of the costs and benefits of nutrition investments similar to those available for some sub‐Saharan countries do not exist for South Asia. The one analysis in Bangladesh estimated that the $1.3–1.7bn cost of implementing a comprehensive package of interventions to improve health status of child under 2 years of age was much smaller than the $10bn loss to GDP because of productivity losses resulting from inaction (Howlader *et al*. 2012). Quality cost‐effectiveness studies for additional countries are needed to build the body of evidence needed to influence policymakers. The existing evidence from other regions has not been enough to expand programmes to improve early childhood nutrition.

Scaling up preventive interventions that include micronutrient supplements for both children and pregnant women is particularly important in the context of South Asia, which is home to persistently high levels of micronutrient deficiencies (iron, vitamin A and iodine). Coverage rates of micronutrient supplement programmes are low: only 24% of pregnant women take iron–folic acid supplements during pregnancy; only 68% of children age 6–59 months receive vitamin A supplementation; and only 70% of the South Asia population uses iodized salt (International Food Policy Research Institute 2014). Similarly, the prevalence of low birthweight (LBW; a birthweight of less than 2.5 kg) is higher in Asia than elsewhere, predominantly because of undernutrition of the mother prior to and during pregnancy and/or being underage (<18 years old) when entering marital union and/or pregnancy. An estimated 25–30% of all infants in South Asia are born with LBW, accounting for half of all LBW babies in the developing world (UNICEF *et al*. 2015).

Although the results reported here are limited to ‘nutrition‐specific’ interventions delivered through the health sector, multisectoral ‘nutrition‐sensitive’ actions through agriculture sector, social protection, water and sanitation and poverty reduction programmes have the potential to strengthen nutritional outcomes in several ways (Ruel *et al*. 2013; World Bank 2013). These interventions address malnutrition in more indirect ways, and there is currently very limited guidance on costing for nutrition‐sensitive interventions for at least two reasons. First, evidence on effectiveness of ‘nutrition‐sensitive’ interventions with respect to nutritional outcomes is limited. 8There are two schools of thought as to why there is currently very little evidence. Some believe that there is little evidence of impact of agricultural interventions on nutrition outcomes because indeed they have no effect. This is referred to as the ‘agriculture–nutrition disconnect’. Another school of thought is that very few agricultural interventions have been designed such as to have an impact on nutrition and so the lack of evidence is inevitable. However, if these interventions were indeed designed to have an impact on nutrition, they could become critical mechanisms for building the evidence base for nutrition‐sensitive programming. Second, compared with nutrition‐specific interventions, estimating and attributing the costs of nutrition‐sensitive interventions is more complex because these interventions have multiple objectives, improved nutrition outcomes being only one of them. Notwithstanding these limitations, the availability of costing information is crucial to assess cost‐effectiveness of these interventions and is needed to engage other sectors in planning for nutritional effects.

## Conclusions

Despite overwhelming evidence of the debilitating costs of stunting, minimal resources are allocated to stunting reduction in South Asia, and where resources are allocated, these are often not focused on scaling up the most cost‐effective interventions. This is largely because stunting in South Asia continues to be invisible to and unrecognized by families, communities and especially policymakers. An appreciation of the economic costs described here can help to make stunting a more ‘visible’ challenge to development. Rigorous estimations of the costs, benefits and cost‐effectiveness of nutrition investments need to be an important next step in all South Asian countries in order to drive political commitment and action and to maximize allocative efficiencies. Knowing how many interventions would cost to scale‐up, what results these might deliver, and how to maximize the effectiveness of the investments provides governments with guidance to prioritize the most cost‐effective interventions as well as with cost‐effective ways of scaling up the interventions over time, thereby maximizing both technical and allocative efficiencies. Furthermore, these rigorous analyses are necessary to leverage new financing from both domestic budgets and overseas development aid.

**Key messages**

- South Asia is home to the largest number of children stunted worldwide.
- Stunting leads to increased mortality and morbidity, poor educational outcomes, lost earnings and reductions in national economic productivity, yet nutrition investments are among the most cost‐effective development actions.
- Analysis of four sub-Saharan countries finds that the scaling up of a set of 10 key nutrition interventions is highly cost-effective and most are very cost‐effective when considered individually.
- Rigorous estimations of the costs and benefits of nutrition investments are an important next step in all South Asian countries in order to drive political commitment and action.

## Conflicts of interest

The authors declare that they have no conflicts of interest.

## Contributions

MS conceptualized and led the project. MS, JDE and JK designed the research. JDE and JK conducted the analysis and wrote the paper. All authors edited and approved the final paper.
